# Leptin: A Novel Therapeutic Target in Alzheimer's Disease?

**DOI:** 10.1155/2012/594137

**Published:** 2012-01-02

**Authors:** Dayne Beccano-Kelly, Jenni Harvey

**Affiliations:** Division of Neuroscience, Medical Research Institute, Ninewells Hospital and Medical School, University of Dundee, Dundee DD1 9SY, UK

## Abstract

It is well established that the hormone leptin circulates in the plasma in amounts proportional to body fat content and it regulates food intake and body weight via its actions in the hypothalamus. However, numerous studies have shown that leptin receptors are widely expressed throughout the CNS and evidence is growing that leptin plays a role in modulating a variety of neuronal processes. In particular, recent studies have highlighted a potential cognitive enhancing role for leptin as it regulates diverse aspects of hippocampal synaptic function that are thought to underlie learning and memory processes including glutamate receptor trafficking, dendritic morphology, and activity-dependent synaptic plasticity. Characterisation of the novel actions of leptin in limbic brain regions is providing valuable insights into leptin's role in higher cognitive functions in health and disease.

## 1. Introduction

The hormone leptin plays a pivotal role in regulating a number of hypothalamic driven functions including energy homeostasis, reproductive function, and bone formation. However, recent studies have demonstrated that leptin has widespread action in the CNS, and evidence is growing that leptin has the capacity to modulate higher brain functions. Indeed, leptin has a marked effect on hippocampal-dependent function and in particular learning and memory processes. In addition, dysfunctions in the leptin system have recently been linked to neurodegenerative disorders such as Alzheimer's disease. Here we review the evidence that leptin is a potential cognitive enhancer and also examine the possibility of utilising leptin replacement therapy in the treatment of Alzheimer's disease.

## 2. Leptin 

The endocrine hormone leptin is principally, but not exclusively, derived from white adipose tissue. The circulating levels of this hormone vary during the day, but are mainly determined by body fat content and also feeding status [1, 2]. Leptin enters the brain via saturable transport across the blood brain barrier [3]. Additionally, leptin may be released locally within the CNS as there is evidence for expression of leptin mRNA and protein in specific neuronal populations [4]. It is well documented that leptin plays a pivotal role in the regulation of food intake and body weight via signaling information about the status of fat stores to leptin receptors expressed on specific hypothalamic nuclei. However, leptin receptors are also widely expressed throughout the CNS with high levels detected in many brain regions involved in higher cognitive processes including the hippocampus, cortex, and amygdala. Moreover, growing evidence indicates that leptin is a pleiotropic hormone that exhibits diverse central actions including its ability to regulate hippocampal synaptic plasticity [5] and to play a role in mood disorders such as depression [6]. 

### 2.1. Leptin Receptors

Leptin mediates its biological effects via activation of the leptin receptor (ObR) which is encoded by the diabetes (*db*) gene [7]. Alternative splicing of the *db* gene results in the generation of six leptin receptor isoforms (ObRa-f) with identical N-terminal binding domains but distinct C-terminal regions and signaling capacity. ObRbis, the long form of the receptor, and the main signaling competent isoform as key motifs required for signaling are contained within its extended C-terminal domain. ObRs display the greatest homology with the class I cytokine receptor superfamily [7]: receptors that lack intrinsic tyrosine kinase activity but signal via association with janus tyrosine kinases (JAKs). Indeed ObR activation results in the phosphorylation of JAK2 which subsequently promotes the association with and activation of various signaling molecules including PI 3-kinase (phosphoinositide 3-kinase), Ras-Raf-MAPK (mitogen activated protein kinase), and STAT3(signal transducer and activator of transcription). 

### 2.2. Leptin Regulation of Hippocampal Synaptic Plasticity

The hippocampal formation is an area of the brain that plays a pivotal role in learning and memory. Indeed, both long-term potentiation (LTP) and long-term depression (LTD), which are activity-dependent forms of synaptic plasticity that result in persistent alterations in excitatory synaptic strength, and which are thought to underlie certain aspects of learning and memory, are evident in this brain region. Moreover, N-methyl-D-aspartate (NMDA) receptor-dependent LTP induced in the hippocampal CA1 region has been implicated in spatial learning and memory. Several lines of evidence indicate that many growth factors and hormones, including insulin [8, 9] and leptin [10]; have the ability to modulate hippocampal synaptic plasticity. Indeed, leptin insensitive obese rodents (*fa/fa* rats and *db/db* mice) display deficits in hippocampal LTP and long-term depression (LTD; [11]). Leptin insensitivity also results in impairments in spatial learning and memory tasks performed in the Morris water maze [11, 12]. Furthermore, rodent performance in spatial memory tasks is significantly enhanced after direct administration of leptin into the CA1 region of the hippocampus, whereas leptin administration into the dentate gyrus increases the magnitude of LTP [13]. At the cellular level, leptin promotes conversion of short-term potentiation (STP) into LTP, and it facilitates the induction of LTP in acute hippocampal slices [13, 14]. 

### 2.3. Leptin Regulates NMDA Receptor Function

It is well established that the synaptic activation of NMDA receptors coupled with a postsynaptic rise in intracellular Ca^2+^ is crucial for the induction of LTP at hippocampal CA1 synapses [15]. Moreover, the ability of hormones to influence the magnitude of LTP predominantly results from modification of NMDA receptor function. Indeed, leptin facilitation of NMDA responses underlies its effects on hippocampal LTP as this hormone enhances both NMDA receptor-dependent synaptic currents in slices and Ca^2+^ influx via NMDA receptor channels in cultured neurons [14]. Studies in *Xenopus* oocytes expressing recombinant NMDA receptors indicate that leptin receptor-driven signalling is required for enhancement of NMDA receptor-mediated currents by leptin. Furthermore, leptin increased maximal NMDA receptor-mediated currents without altering channel kinetics, suggesting that leptin increases the number of functional NMDA receptor channels by boosting NMDA receptor trafficking to the cell membrane. 

In contrast to *α*-amino-3-hydroxy-5-methylisoxazole-4-propionic acid (AMPA) receptors that readily move to and away from synapses as well as laterally within the plasma membrane [16, 17], NMDA receptors were viewed, until fairly recently, as static entities. However, there is growing evidence that the molecular identity and number of synaptic NMDA receptors can be modulated in an activity-dependent manner and in response to sensory experience [18]. For instance, the induction of LTP at adult hippocampal CA1 synapses is accompanied by an increase in NMDA receptor surface expression [19]. In addition, hormones such as insulin and the extracellular matrix protein reelin have been shown to modify NMDA receptor trafficking processes [20, 21]. NMDA receptors are heteromeric assemblies of NR1 and NR2 subunits, with or without an NR3 subunit, and the NR2 subunits determine the biophysical and pharmacological properties of NMDA receptors [22]. The expression and localization of NR2 subunits changes during development. Moreover the polarity of hippocampal synaptic plasticity depends on NMDAR subunit composition at different developmental stages. In adult hippocampus NR2A subunits underlie LTP whereas NR2B subunits are implicated in LTD [23]. In contrast, NR2A and NR2B underlie LTP, and LTD is NR2B independent in juvenile hippocampus [24]. Recent studies indicate that the intracellular C-terminal region of NR2 subunits plays a central role in controlling trafficking of NMDA receptors. Moreover, phosphorylation and palmitoylation of NR2C-terminal domains are also key events regulating NMDA receptor trafficking [25, 26]. Previous studies have shown that leptin has the ability to increase NR1/NR2A-mediated currents in *Xenopus* oocytes; an effect that is likely to be due to increased trafficking of NMDA receptors to the plasma membrane [5]. However no studies to date have examined if leptin alters the trafficking of other NR2 subunits, and thus it remains to be established if this hormone regulates NMDA receptors in an NR2-dependent manner.

### 2.4. Leptin Promotes Trafficking of GluR2-Lacking AMPA Receptors to Synapses

Previous studies indicate that application of leptin to juvenile (2-3 weeks old) hippocampal slices results in a transient depression of excitatory synaptic transmission [14, 27]. Conversely, leptin evokes a robust enhancement of excitatory synaptic transmission in adult hippocampus, and this effect persists after leptin washout [28]. Activation of leptin receptors is necessary for the leptin-driven increase in excitatory synaptic strength as leptin was without effect in slices from leptin-insensitive Zucker *fa/fa* rats, but a robust effect of leptin was observed in age-matched Zucker lean animals. In addition, the leptin increase in synaptic transmission was not associated with any significant change in PPR or CV indicating a likely postsynaptic expression mechanism. It is well documented that NMDA receptor activation is pivotal for LTP induction [29], and activation of NMDA receptors underlies the ability of leptin to facilitate LTP induction, reverse established LTP, and promote changes in dendritic morphology [14, 29, 30]. Similarly, leptin failed to increase excitatory synaptic strength in slices perfused with the NMDA receptor antagonist D-AP5, indicating the involvement of an NMDA receptor-dependent process. Moreover, in two pathway experiments leptin had no effect when stimulation was stopped whereas leptin significantly increased synaptic transmission in the control pathway indicating that the synaptic activation of NMDA receptors was required. 

It is known that NMDA receptor activation underlies the trafficking of AMPA receptors to hippocampal synapses during LTP [31], and that changes in the subunit composition of synaptic AMPA receptors are linked to activity-dependent changes in synaptic efficacy [3]. AMPA receptors are heterotetrameric ion channels composed of GluR1-4 subunits. GluR2-lacking AMPA receptors are more important for hippocampal LTP than GluR2-containing AMPA receptors, due to their permeability to Ca^2+^ which in turn allows for the activation of specific intracellular signalling pathways required for long-term changes in synaptic efficacy [32, 33]. Neurons within the hippocampus predominantly express functional GluR2-containing AMPA receptors until times of increased synaptic activity, when the membrane localized complement alters to include more GluR2-lacking AMPA receptors. Recent studies indicate that alterations in AMPA receptor trafficking processes also contribute to the increase in synaptic efficacy induced by leptin. Indeed, in studies examining the rectification properties of synaptic currents, the leptin-driven increase in synaptic transmission was accompanied by an increase in AMPA receptor rectification indicating that an increase in the synaptic density of GluR2-lacking AMPA receptors underlies this effect. Moreover, application of philanthotoxin resulted in reversal of the leptin-driven increase in synaptic transmission which is also consistent with an increase in the GluR2-lacking AMPA receptors underlying the increase in synaptic efficacy induced by leptin. In parallel studies, the effects of leptin on the cell surface expression of GluR1 and GluR2 in acute hippocampal slices and hippocampal cultures were examined using biotinylation assays and immunocytochemistry, respectively [28]. In adult hippocampal slices leptin enhanced GluR1, but not GluR2, surface expression. Conversely leptin failed to alter the surface expression of either GluR1 or GluR2 in slices from younger animals (3-4 week old). Furthermore, in dual immunolabeling experiments leptin was circa 50fold more potent at increasing GluR1 relative to GluR2 surface expression in hippocampal cultures. The ability of leptin to increase GluR1 surface expression involves promotion of GluR1 exocytosis as the effects of leptin were blocked by inhibitors of exocytosis, namely, NEM (N-ethylmaleimide-sensitive fusion protein) and brefeldin A. Consistent with this, the leptin-dependent enhancement of excitatory synaptic transmission in adult hippocampal slices was prevented following whole cell dialysis with exocytotic (NEM and brefeldin A), but not endocytotic (bafilomycin), inhibitors. 

### 2.5. Role of PTEN in Leptin-Driven AMPA Receptor Trafficking to Synapses

Previous studies have demonstrated that PI 3-kinase, an enzyme that phosphorylates PtdIns(4,5)P_2_ into PtdIns(3,4,5,)P_3_, plays a pivotal role in leptin-driven signaling in the hippocampus [10]. PI 3-kinase is also implicated in NMDA receptor-dependent AMPA receptor trafficking to synapses during hippocampal LTP [31]. Similarly a PI 3-kinase-driven process underlies the effects of leptin as the increase in GluR1 surface expression was correlated with enhanced levels of PtdIns(3,4,5,)P_3_ immunostaining, suggesting that an increase in PtdIns(3,4,5,)P_3_ levels underlies leptin-driven alterations in AMPA receptor trafficking. Additionally blockade of PI 3-kinase signaling with either wortmannin or LY294002 prevented both the increase in excitatory synaptic transmission and GluR1 surface expression induced by leptin. In support of a possible role for PtdIns(3,4,5,)P_3_, enhanced PtdIns(3,4,5,)P_3_ synthesis results in significant enhancement of AMPA, but not NMDA, receptor-mediated synaptic transmission [34]. Although these findings suggest the involvement of a PI 3-kinase-dependent process, PtdIns(3,4,5,)P_3_ levels are also regulated by the phosphatase PTEN as it antagonizes PI 3-kinase activity by catalysing the conversion of PtdIns(3,4,5,)P_3_ to PtdIns(4,5)P_2_ [35]. Moreover PTEN has been identified as a key signaling pathway activated by hypothalamic leptin receptors, and leptin receptor activation of K_ATP_ channels involves phosphorylation and subsequent inhibition of PTEN [36, 37]. In support of a role for PTEN, exposure of hippocampal slices to leptin increased the phosphorylation of PTEN and this effect was absent in slices from Zucker *fa/fa* animals indicating the involvement of a leptin receptor-driven process. Furthermore, the increase in GluR1 surface expression induced by leptin was coupled with an increase in P366-PTEN immunostaining in hippocampal cultures [28]. Previous studies have shown that CK2 phosphorylates PTEN at the threonine 366 site [38]. In agreement with these studies, the ability of leptin to increase GluR1 surface expression, P366-PTEN phosphorylation and excitatory synaptic transmission were all blocked by casein kinase2 (CK2) inhibition. This in turn supports the notion that CK2 phosphorylation and subsequent inhibition of PTEN underlies leptin-driven alterations in AMPA receptor trafficking and excitatory synaptic strength.

GluR1 surface expression was also significantly increased in neurons transfected with dominant-negative PTEN mutants (C124S or G129E). However, the ability of leptin to increase GluR1 surface expression was occluded in cells expressing the PTEN mutants, suggesting that analogous mechanisms underlie both processes. Leptin also increased the amplitude but not the frequency of mEPSCs, an effect attributable to insertion of GluR2-lacking AMPA receptors as it was reversed by addition of philanthotoxin. In contrast leptin failed to alter mEPSC amplitude in neurons transfected with the PTEN mutants. Thus inhibition of PTEN not only increases the functional expression of GluR1 subunits at hippocampal synapses but it also prevents the trafficking of GluR1 subunits by leptin. Similarly, pharmacological inhibition of PTEN with the phosphatase inhibitor bisperoxovanadium (bpV; [39]) resulted in a persistent increase in excitatory synaptic transmission in hippocampal slices, and it increased trafficking of GluR1 to hippocampal synapses. In addition, leptin failed to enhance synaptic transmission or alter AMPA receptor trafficking in the presence of bpV, which further supports the notion that leptin increases the synaptic expression of GluR1 via inhibition of PTEN and subsequent increase in PtdIns(3,4,5,)P_3_ levels. However it is not exactly known how elevations in PtdIns(3,4,5,)P_3_ levels modify AMPA receptor trafficking processes. Recent studies have shown that the availability of PtdIns(3,4,5,)P_3_ is pivotal for sustaining AMPA receptor clustering and synaptic function at hippocampal synapses [34]. As inositol lipids are important regulators of the actin cytoskeleton, PtdIns(3,4,5,)P_3_ may influence AMPA receptor trafficking by rearranging the actin cytoskeleton [40]. Alternatively, PtdIns(3,4,5,)P_3_ may stimulate the activation of the protein kinase, Akt, which in turn may phosphorylate and subsequently inhibit glycogen synthase kinase 3 (GSK-3). In support of this possibility, Akt-driven inhibition of GSK-3 underlies AMPA receptor insertion after hippocampal LTP [41]. The ability of leptin to rapidly alter AMPA and NMDA receptor trafficking processes and evoke persistent changes in excitatory strength provides further evidence to support a role for this hormone as a potential cognitive enhancer. The leptin receptor-driven alterations in hippocampal synaptic function are likely to play an important role not only in normal brain function, but also in CNS-driven diseases associated with leptin dysfunction.

## 3. Leptin and Aging

Several lines of evidence support the notion that the effectiveness of metabolic hormonal systems declines with age and that impaired energy metabolism not only accelerates the aging process but also increases the susceptibility to neuronal degeneration [42]. Numerous studies have examined how insulin signaling in the CNS is altered during aging, but our understanding of how the leptin system changes with age is limited. A recent study comparing the effects of leptin on excitatory synaptic transmission in hippocampal slices from 3-4 month- and 12–14 month-old animals found that the responsiveness of hippocampal CA1 neurons to leptin declines with age [43]. In accordance with previous studies [28], leptin resulted in a persistent increase in the efficacy of hippocampal excitatory synaptic transmission (leptin-induced LTP) at 12–14 months, however, the magnitude of increase was significantly less at this age compared to 3-4 months. It is known that the magnitude of hippocampal LTP attenuates with age and this has been linked to reduced activation of NMDA receptors during the induction of LTP [44, 45]. It is feasible that a reduction in NMDA receptor activation contributes to the fall in the magnitude of leptin-induced LTP with age, as the ability of leptin to induce LTP also requires the synaptic activation of NMDA receptors and leptin-induced LTP and synaptically induced LTP share similar expression mechanisms [43]. Indeed, the magnitudes of LTP induced by leptin and high frequency stimulation were analogous in both adult and aged hippocampus, and synaptically induced LTP occluded the persistent increase in synaptic transmission induced by leptin and vice versa [43]. Although this study provides good evidence for a decline in hippocampal leptin function with age, it is not yet clear if the ability of leptin to modulate other CNS functions is altered during the aging process. 

## 4. Leptin and Alzheimer's Disease

As life expectancy is increasing steadily, the prevalence of age-related neurodegenerative disorders such as AD is also increasing. Our understanding of the cellular changes that occur in the early stages of AD has advanced significantly in recent years, but it is still extremely difficult to uncover these early aberrant changes in a clinical setting. It is known that various factors can enhance the risk of developing AD including lifestyle and diet. Moreover, several studies have highlighted an association between midlife obesity and the incidence of AD, however, the mechanisms underlying this association are unclear. A number of studies have proposed that leptin dysfunction provides a link between obesity and AD [46]. Indeed, it is known that obesity is triggered by elevated leptin levels and the subsequent development of leptin resistance. In support of a possible link between obesity and AD, the circulating levels of leptin are significantly lower than normal in AD patients [47]. In addition, recent studies have shown that individuals with higher leptin levels have a much lower risk of developing AD [48, 49]. Moreover, leptin levels are also significantly reduced in murine models (APP_Swe_; PSI_M146V_) of AD [50]. 

Previous studies have indicated that leptin has neuroprotective and antiapoptotic properties as it protects neurons from a variety of neurotoxic agents including TNF*α*, ferrous iron (Fe^2+^), and 6-OHDA [51–53]. Recent studies also support a neuroprotective role for leptin against ischaemic episodes [54]. These findings have important implications for the role of leptin in neurodegenerative disorders, as ischaemic incidents (such as cerebral thrombosis or stroke) have been shown to increase the incidence of sporadic AD by as much as 10-fold [55, 56]. Leptin has also been shown to have proliferative effects on neurones [57] increasing hippocampal volume and neuronal progenitor number, as well as reducing neurodegeneration caused by AD-related mutations [57]. Several studies have shown that leptin has neurotrophic actions in the CNS, however, this may be restricted to specific neuronal populations as leptin promotes neurite outgrowth in cerebellar purkinje, but not granule cells [58]. Further support for a neurotrophic role comes from a study by Yamada et al. [59] that demonstrated that leptin could alter cognitive state by reducing depression measured as a function of despair response in mice. Furthermore, leptin increased the levels of BDNF in the hippocampus, resulting in direct and inverse effects on the depressed state of the mice [59]. These findings lend additional support to the notion that leptin treatment could be beneficial in AD which is associated with neurodegeneration and cognitive impairments such as depression and dementia.

It is well established that two key pathological hallmarks of AD are the formation of amyloid plaques, due to the build-up and accumulation of *β*-amyloid (A*β*) and neurofibrillary tangles resulting from hyperphosphorylation of tau. Thus it is feasible that leptin limits the toxic effects of A*β* in neurons. Indeed, leptin is reported to attenuate A*β* levels in neurons by inhibiting *β*-secretase activity and thereby reducing A*β* production [60]. Furthermore, leptin promotes ApoE-driven uptake of A*β* into neurons [60]. Leptin also has the capacity to alter the levels of hyperphosphorylated tau as leptin reduces the accumulation of phosphorylated tau in neuronal cells [61] and it limits phosphorylation of tau by inhibiting GSK3*β* [61]. Treatment of murine models of AD with leptin also resulted in significant reductions in the levels of both A*β* and phosphorylated tau compared to vehicle-treated littermates [62]. In addition to ameliorating AD pathology, treatment of CRND8 transgenic mice with leptin resulted in enhanced performance in novel object recognition tests as well as contextual and cued fear conditioning [63]. In SAMP8 mice, with elevated A*β* levels, administration of leptin improved memory processing in the T-maze foot-shock avoidance and step-down inhibitory avoidance tests [63]. Thus together these findings indicate that leptin has the ability to not only reduce the toxic accumulation of A*β* and phosphorylated tau but it also improves memory in murine models of AD. 

Although numerous studies indicate that leptin markedly influences CNS function in rodent models, to be an effective therapeutic agent in CNS-driven disease leptin must have the capacity to modulate human brain function. Recent clinical studies have shown that treatment of three adults, with congenital leptin deficiency, with physiological doses of leptin resulted in significant and persistent elevations in grey matter volume in specific regions of the brain including the cerebellum and anterior cingulated gyrus [64]. Another study found evidence that leptin replacement therapy influences cognitive function, as treatment of a five-year-old boy with congenital leptin deficiency not only restored normal body weight and glycemic control, but also significantly improved neurocognitive skills [65]. 

## 5. Conclusions

Evidence is accumulating that the hormone leptin has widespread actions in the brain and it has the ability to regulate numerous CNS functions. In particular, evidence is accumulating that leptin plays a pivotal role in modulating higher cognitive functions such as learning and memory. Indeed, recent studies indicate that leptin is a potential cognitive enhancer as it rapidly alters glutamate receptor trafficking processes and in turn the efficacy of hippocampal excitatory synaptic transmission. However, the effects of leptin on hippocampal synaptic function markedly decline with age. Obesity in humans is closely associated with development of type II diabetes, and it is well documented that cognitive deficits are prevalent in diabetics. As obesity and obesity-linked disorders such as type II diabetes are associated with resistance to leptin, it is feasible that leptin dysfunction plays a role in cognitive impairments in these individuals. In addition, recent studies have linked dysfunctions in the leptin system with the development of neurodegenerative disorders such as Alzheimer's disease. Moreover, growing evidence indicates that leptin prevents the toxic accumulation of A*β* and phosphorylated tau in neurons, and it has the ability to improve performance in various memory tasks in murine AD models. These findings, coupled with the already established safety of leptin in humans, make this hormone or related leptin-mimetics novel therapeutic candidates for the treatment of neurodegenerative disorders like AD. 
